# Comparison of bacteria disintegration methods and their influence on data analysis in metabolomics

**DOI:** 10.1038/s41598-021-99873-x

**Published:** 2021-10-21

**Authors:** Karolina Anna Mielko, Sławomir Jan Jabłoński, Marcin Łukaszewicz, Piotr Młynarz

**Affiliations:** 1 Department of Biochemistry, Molecular Biology and Biotechnology, Faculty of Chemistry, Wroclaw University of Science and Technology, Wroclaw, Poland; 2 Biotransformation Department, Faculty of Biotechnology, University of Wroclaw, Wroclaw, Poland

**Keywords:** Microbiology, Small molecules

## Abstract

Metabolomic experiments usually contain many different steps, each of which can strongly influence the obtained results. In this work, metabolic analyses of six bacterial strains were performed in light of three different bacterial cell disintegration methods. Three strains were gram-negative (*Pseudomonas aeruginosa*, *Escherichia coli,* and *Klebsiella pneumoniae*), and three were gram-positive (*Corynebacterium glutamicum*, *Bacillus cereus, *and *Enterococcus faecalis*). For extraction, the methanol–water extraction method (1:1) was chosen. To compare the efficiency of different cell disintegration methods, sonication, sand mill, and tissue lyser were used. For bacterial extract metabolite analysis, ^1^H NMR together with univariate and multivariate analyses were applied. The obtained results showed that metabolite concentrations are strongly dependent on the cell lysing methodology used and are different for various bacterial strains. The results clearly show that one of the disruption methods gives the highest concentration for most identified compounds (e. g. sand mill for *E. faecalis* and tissue lyser for *B. cereus).* This study indicated that the comparison of samples prepared by different procedures can lead to false or imprecise results, leaving an imprint of the disintegration method. Furthermore, the presented results showed that NMR might be a useful bacterial strain identification and differentiation method. In addition to disintegration method comparison, the metabolic profiles of each elaborated strain were analyzed, and each exhibited its metabolic profile. Some metabolites were identified by the ^1^H NMR method in only one strain. The results of multivariate data analyses (PCA) show that regardless of the disintegration method used, the strain group can be identified. Presented results can be significant for all types of microbial studies containing the metabolomic targeted and non-targeted analysis.

## Introduction

The analysis of cell metabolite compositions and concentrations (metabolomics) is a rapidly developing research tool. It was successfully used to analyze bacterial cell adaptation, microorganism identification, and phage infection mechanisms^1^. It is also considered a promising diagnostic tool in the case of bacterial infections^2^. Analytical technologies used in metabolomics include mainly chromatography coupled with mass spectrometry (MS) or nuclear magnetic resonance (NMR).

Due to the low concentration of metabolites (approximately 2% of cell dry mass) and detection limits of analytical techniques, the investigation of intracellular metabolites usually requires initial extraction and concentration. The metabolite extraction efficiency determines the amount of biomass required for the experiment, which may cause problems due to low biomass yields for certain species of microorganisms^3,4^.

Methods used in sample preparation differ depending on reported research (Table 1). This situation may be confusing for scientists starting their adventure with metabolomics. Moreover, it was proven that the choice of sample preparation method may influence obtained metabolite profile^5^. Thus making comparisons of data obtained by different research teams is very difficult.

**Table 1:** Cell disruption methods used in metabolome analysis.

Disruption method	Extraction method	Organism	Amount of biomass	Analytical method	References
Freeze–thaw (× 3)	Chloroform/methanol/water (1:3:1)	*P. aeruginosa*	∼10^8^ CFU/ml (OD_600_ = 0.5)	HPLC/LC–MS	^6^
Ultrasonic bath 15 min. 70 °C	Methanol/water/chloroform (3:3:2)	*P. aeruginosa*	150 mg wet biomass	GC/MS	^7,8^
60% ethanol at 78 °C for 2 min, liquid nitrogen freezeing	3 ml ethanol (60%)	*P. aeruginosa*	4·10^8^ CFU, 1 ml OD600 1.0	TOF–MS	^9^
Vortexed with methanol	Methanol/water/chloroform (5:5:8)	*P. aeruginosa*	300 mg wet biomass	^1^H NMR	^10^
Freeze–thaw (× 3) in 50% methanol	Methanol/water (1:1)	*K. pneumoniae*	∼8·10^8^ CFU/ml (OD_550_ = 0.7)	^1^H NMR	^11^
Cryostat (∼ − 50 °C)	100% methanol	*K. pneumoniae*	(OD_600_ = 0.4–0.6)	LC/MS	^12^
Homogenization with PBS and sonication bath 30 min	PBS buffer	*K. pneumoniae*	300 ml, OD_600_ = 0.7–0.9	^1^H NMR	^13^
Freeze–thaw (× 3) in 50% methanol, liquid nitrogen freezeing 1 min	Methanol/water (1:1)	*B. cereus*	50 mg	GC/TOF–MS	^14,15^
Boiling in water for 15 min	Water	*C. glutamicum*	1–4 mg	GC/MS	^16^
Ultrasonic bath 15 min 70 °C in methanol	Methanol/water/chloroform (3:3:2)	*C. glutamicum*	5·10^10^ CFU	GC/MS	^17^
Incubation with solvents in − 20 °C for 4 h	Methanol/water/chloroform (1:1:2)	*C. glutamicum*	20–50 mg wet biomass	LC/MS–MS	^18^
Freeze–thaw (×3) in 50% methanol	Methanol/water (1:1)	*E. faecalis*	50 ml of culturebroth	GC/MS	^19^
Sonication: sequence (6 s/4 s) for 6 min and bath for 20 min	Methanol/water/chloroform (4:1:1)	*E. coli*	5 ml, OD_600_ = 1.0	GC–MS	^20^
Freeze–thaw (×3) in methanol, liquid nitrogen freezeing	100% methanol	*E. coli*	20 ml, 10^8^ CFU/ml	^1^H NMR	^21^

Choosing a proper disruption method and conditions for selected materials may be crucial for the reliability of the obtained experimental results. “Too-mild” conditions lead to a lower metabolite extraction efficiency and underrepresentation of metabolites from more break-up-resistant cells. This effect may be very significant for samples containing different species of microorganisms. On the other hand, an excessively long disintegration process may alter the metabolite composition due to the degradation of liable compounds or enzymatic reactions^4,10^.

The bacterial cytoplasmic membrane is the most important barrier holding metabolites in the cell. It may be passively passed by small uncharged or nonpolar molecules (water, carbon dioxide or hydrogen, protonated organic acids). The membrane is relatively susceptible to disruption with chemical agents such as organic solvents or detergents^22,23^, and it is not an effective barrier for hydrophobic molecules. Due to lipid solubility in organic solvents, most metabolite extraction protocols use organic solvents such as methanol, chloroform, or ethanol. The additional role of organic solvents is the denaturation of enzymes, which may influence the metabolite profile after cell disruption.

The presence of a thick cell wall could reduce the amount of extracted metabolites. The cell wall is resistant to disintegration with chemical solvents, however, it is not as an effective barrier for soluble molecules as lipid membrane. Passive diffusion through the cell wall is possible for globular molecules up to 25 kDa^24^. It is known that cell wall disruption affects the metabolomic profile obtained of bacteria and the effect is much stronger in the case of gram-positive *Enterococcus faecalis* than for gram-negative *Escherichia coli*^22,24^.

The more substantial effect of cell wall disruption in the case of *E. faecalis* may be explained by bacterial cell wall resistance and structure. Bacteria are classified as gram-positive or gram-negative. This classification originates from the result of Gram staining, which is associated with the structure of the cell wall. The bacterial cell wall is mainly composed of the peptidoglycan polymer, which is also known as murein. In the case of gram-negative bacteria, the layer of peptidoglycan is localized in the periplasmatic space and is relatively thin. In *Escherichia coli* the peptidoglycan layer is flexible net, and its thickness is between 2.5 and 6 nm^25^. The pressure required for the destruction of the cell wall in *E. coli* is around 50 MPa^26^. In gram-positive bacteria, only one lipid membrane is present, and the outer peptidoglycan layer is thicker. In species like *Staphylococcus aureus* murein still resembles a net composed of relatively short amino sugar strands (6 disaccharide units on average)^27^. The thickness of this structure is around 25 nm^28^. In the case of *Bacillus subtilis* the cell wall organization is more sophisticated. The cell wall is composed of long murein cables wrapped around the cell along the longer axis. A considerable fraction (around 25%) of peptidoglycan strains is longer than 500 disaccharide units^29^. The cell wall of gram-positive species, in general, is regarded as tough. The pressure required to destroy the cell wall in *Staphylococcus aureus* is around 250 MPa, and in *B. subtilis* it is around 100 MPa, respectively. Higher durability of *S. aureus* cell may result also from spherical cell shape. Cell resistance in the general population of bacteria is not equal for all cells. Thus disruption of 50% of cells is much easier than disrupting 95% of cells^26^.

Several methods were developed to achieve this since disruption of the bacterial cell wall is crucial in many laboratories and industrial operations (DNA and protein isolation). Physical cell disruption methods involve the following processes: pressure disruption, sonication (exposure to ultrasound), freezing, and milling^30,31^. In pressure disruption, cells are forced to pass through narrow channels with high flow velocities. The cells are disrupted by forces caused by shear stress, turbulence, and friction. During sonication, cells are disrupted by shock waves produced by a dedicated device. During milling, cells are squeezed and damaged during collisions with bead particles and vessel walls. Freezing causes the formation of water crystals inside cells, resulting in volume extension and cell disruption. The efficiency of the cell disruption process depends on the amount of energy delivered to the system. Better disruption requires harsh conditions or longer time^26,30^.

In metabolomics studies, each step of the sample preparation influences results. An adequately prepared protocol is more reliable and can be useful. Metabolomics as a scientific branch could give information about differences between microorganisms. The most popular analytical tool in clinical laboratories is mass spectrometry (MS)^32^, but the results of previous experiments provided evidence that the nuclear magnetic resonance (NMR) method can be used as an analytical tool for rapid bacterial identification^33^. Metabolic analysis has been used to examine and compare extra- and intracellular bacterial primary and secondary metabolites. Furthermore, it can be useful in pathway discovery and regulation^34^. These examples clearly show that the development of NMR techniques, database creation, and finding the most suitable sample preparation protocol can improve metabolomics studies and probably support future clinical diagnosis.

Our goal was to determine the influence of the cell mechanical disruption method on the metabolite profile obtained with ^1^H NMR spectroscopy for six different bacteria species. Bacteria species selection was based on their difference in cell wall structure and shape. These factors may influence cell mechanical resistance and metabolite extraction procedure efficiency.

## Objectives

The research aimed to check which disintegration method is the best for conducting NMR analysis (metabolomic fingerprinting) and if it influences the analysis of different bacterial strains.

## Material and methods

### Bacterial strains and culture conditions

In this study, six strains were analyzed. Three strains were gram-negative bacteria (*Pseudomonas aeruginosa DSMZ 1707*, *Escherichia coli ATCC 9212*, and *Klebsiella pneumoniae ATCC 700603*), and three were gram-positive bacteria (*Enterococcus faecalis ATCC 29212*, *Bacillus cereus ATCC 11778*, and *Corynebacterium glutamicum ATCC 13287*).

In the first step, the strains were routinely grown on Miller’s LB broth agar (BioShop) with 0.5% glucose (BioShop) (Behrends, 2013), which provided growth suitable for the collection of the inoculum.

To evaluate the differences between strains, bacteria were cultured in 10 cm Petri dishes for 24 h at 37 °C. Afterward, a preculture was prepared by inoculating the bacterial culture into 20 ml of liquid LB broth medium and incubated for 24 h at 37 °C with shaking (315 r.c.f.). Next, 100 ml of the culture was prepared in a 300 ml Erlenmeyer flask. The initial OD_600nm_ for all cultures was 0.1. Cultures were incubated at 37 °C for the appropriate time for the particular strain (Table 2).

**Table 2:** The p-values resulting from analysis of variance between three disintegration methods in each bacteria strain (BC—*B. cereus*; CG—*C. glutamicum*; EF—*E. feacalis*; EC—*E. coli*; KP—*K. pneumoniae*; PA—*P. aeruginosa*).

Metabolite	G(+)			G(−)		
	BC	CG	EF	EC	KP	PA
5-Aminopentanoate	–	–	–	–	–	**2.90E−02**
4-Aminobutyrate	7.57E−01	1.21E−01^#^	**4.94E−03**	7.33E−01^#^	5.39E−02^#^	–
Acetate	1.90E−01	2.72E−01	**8.56E−04**	**8.65E−03**^**#**^	**3.52E−04**	**7.30E−05**
Adenine	2.91E−01^#^	**8.42E−03**	–	–	2.05E−01	–
Adenosine	8.31E−02	–	**9.02E−03**	**1.93E−03**^**#**^	1.14E−01^#^	–
Alanine	**3.43E−03**	7.99E−01	**1.28E−03**	**3.88E−02**^**#**^	**4.24E−02**^**#**^	**5.62E−04**
AMP	–	9.23E−02	5.47E−02^#^	**8.08E−03**^**#**^	–	3.57E−01
Asparagine	–	–	1.17E−01	–	–	–
Aspartate	5.70E−01	6.31E−01	1.05E−01	–	–	3.72E−01
Betaine	7.62E−01	5.19E−01	6.82E−01	–	8.88E−01	8.05E−02^#^
Cholate	5.09E−02	**2.99E−03**^**#**^	1.45E−01^#^	**1.93E−02**^**#**^	**2.53E−02**	**3.65E−02**^**#**^
Formate	**3.48E−02**	**1.17E−07**	1.98E−01	**4.00E−02**^**#**^	9.85E−01^#^	**1.91E−03**^**#**^
Fumarate	–	–	–	–	8.06E−01	–
Glutamate	7.56E−01	4.48E−01	2.33E−01	–	–	2.72E−01
Glutamine	–	–	7.09E−01	–	–	–
Glycine	1.04E−01^#^	6.78E−01	1.37E−01^#^	**9.44E−03**^**#**^	**2.31E−02**	4.02E−01
Histidine	9.21E−01	**5.25E−03**^**#**^	1.40E−01^#^	4.03E−01^#^	6.14E−02	–
Inosine	5.21E−02	**3.54E−02**^**#**^	**1.24E−02**	–	–	–
Isocitrate	–	–	–	–	–	**1.06E−02**^**#**^
Isoleucine	1.90E−01	6.26E−01	1.78E−01	9.97E−02^#^	1.18E−01	8.24E−01
Lactate	3.55E−01	**9.35E−03**	5.41E−01	**1.83E−02**^**#**^	7.66E−01	2.68E−01
Leucine	5.48E−01	1.54E−01	1.82E−01^#^	1.25E−01^#^	2.46E−01	6.43E−02
Lysine	6.97E−01	9.14E−01^#^	**4.93E−03**	**1.28E−02**^**#**^	**3.80E−02**^**#**^	**4.49E−06**
Methionine	**5.90E−05**	**9.63E−03**^**#**^	**3.07E−03**^**#**^	**1.07E−15**^**#**^	**3.68E−03**	**5.92E−04**
NAD+	9.97E−02^#^	**1.79E−02**	2.30E−01^#^	**7.11E−03**^**#**^	3.66E−02	4.62E−01
Nicotinate	–	–	–	–	2.30E−01^#^	–
O-Phosphocholine	**4.58E−02**	2.06E−01	5.93E−01	**2.00E−02**^**#**^	3.20E−01^#^	**5.36E−03**
Oxypurinol	5.48E−02	**8.25E−03**	–	–	4.06E−01	–
Phenylalanine	3.00E−01	9.22E−01	8.05E−01	9.14E−01^#^	4.24E−01	2.95E−01
Propyleneglycol	**7.81E−03**	–	–	–	1.37E−01	–
Pyruvate	4.12E−01	5.08E−01	**4.53E−02**^**#**^	**3.07E−03**^**#**^	3.25E−01^#^	5.78E−01
Sarcosine	**4.47E−03**^**#**^	9.91E−01	**1.10E−02**	–	1.12E−01	1.53E−01
Succinate	6.80E−01	4.16E−01	4.39E−01	6.13E−02^#^	3.68E−01^#^	2.87E−01
Threonine	9.18E−01	4.39E−01	9.05E−01	1.68E−01^#^	6.77E−01^#^	1.38E−01
Trehalose	–	5.10E−01^#^	–	–	–	–
Tyramine	9.05E−01	7.50E−01	5.65E−01	–	2.93E−01	3.53E−01
Tyrosine	4.00E−01	9.22E−01	4.62E−01	–	3.67E−01	5.59E−01
UDP-glucose	7.57E−01	4.63E−01	–	**6.99E−03**^**#**^	–	–
Uracil	5.59E−01	–	–	2.31E−01^#^	9.64E−01	1.45E−01
Uridine	**1.00E−02**	–	–	–	–	–
Valine	1.88E−01	4.48E−01	2.72E−01	5.39E−02^#^	5.31E−02^#^	2.24E−01
β-Alanine	8.99E−01	8.99E−01	–	–	–	–

^#^Kruskal–Wallis test; bold—results with p-value < 0.05.

Growth curve measurements (in triplicate) were conducted for each strain to determine when the bacteria were in a logarithmic growth phase. For this purpose, the absorbance of the samples was measured at a wavelength of 600 mm. The measurement was started during the establishment of the culture (from OD_600 nm_ = 0.1) and was carried out for 16 h. The growth curves are available in the Supplementary materials (Figure S1). These results allowed us to obtain the culturing time for each strain. It was seven hours for *P. aeruginosa*, four hours for *E. coli*, three and a half hours for *K. pneumoniae*, three hours for *E. faecalis*, five hours for *B. cereus*, and nine hours for *C. glutamicum*.

After this time, the cultures were centrifuged (23,635 rcf, 5 min, 4 °C) (Sigma 3-18KS, Polygen), and the bacterial pellet was washed with 0.9% NaCl solution and stored at − 80 °C. To determine the number of cells, the bacterial pellet was lyophilized (ScanvacCoolsave, Labogene). Before extraction, 10 mg of each sample was weighed in tubes (Eppendorf). The entire protocol was repeated for each strain, and each disintegration method was performed five times.

### Extraction, disintegration and samples preparation

Ten milligrams of lyophilized cells were suspended in 500 µl of methanol (LiChrosolv) and 500 µl of water (LiChrosolv). To compare the effectiveness of disintegration methods, we chose three methods. In the first case, sonication was used. The samples were sonicated for 5 min in a 15 s/15 s cycle (Microson Ultrasonic Cell Disruptor, Mison). The second method used a sand mill. For each sample, 0.5 mL of 0.5 mm glass balls (Carl Roth GmbH + Co. KG) was added and homogenized in 9 cycles of 60 s/60 s (FastPrep-24 5G Sample Preparation System, M. P. Biomedicals). In the third method of disintegration, a tissue lyser was used (Tissue Lyser II, Qiagen). Samples were homogenized for 10 min at 30 1/s frequency. Each sample was performed in five replicates.

After disintegration, the samples were centrifuged (2100 rcf, 10 min, 4 °C) (Micro 220R, Hettich), and 720 µl of the clarified upper phase was transferred into a new tube (Bionovo). The extracts were evaporated in a vacuum centrifuge (40 °C, 1500 rpm, 8 h) (WP-03, JWElectronic). In the next step, 600 µl of PBS buffer (0.5 M,10% D_2_O, 1 g NaN_3_, pH = 7.0, TSP = 0.3 mM) was added to each sample and mixed for 3 min. The obtained samples were centrifuged (21,000 rcf, 10 min, 4 °C), and 550 µl was transferred into 5-mm NMRtubes (5SP, Armar Chemicals) for measurement. Until the measurements were taken, the samples were stored at 4 °C.

**Figure 1:**
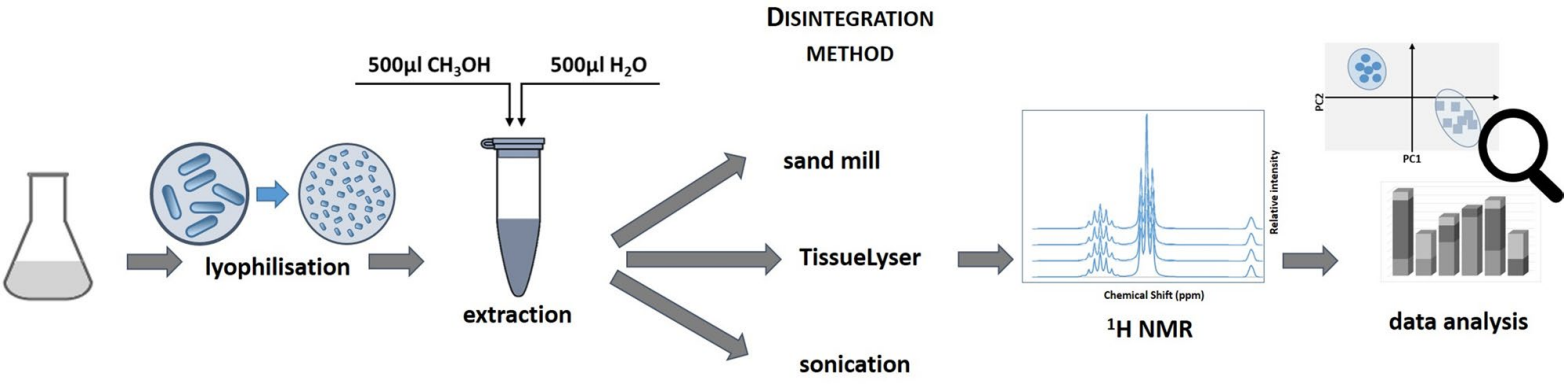
The experiment scheme.

The experimental scheme is shown below (Fig. 1).

### ^1^H NMR spectroscopy analysis of the bacterial metabolites

Standard one-dimensional ^1^H NMR experiments were performed on a Bruker AVANCE II 600.58 MHz spectrometer. All one-dimensional 1H NMR spectra were obtained using the *cpmgpr1d* pulse sequence (in Bruker notation) by the suppression of water resonance through presaturation. The acquisition parameters were as follows: spectral width, 20.01 ppm; the number of scans, 128; relaxation delay, 3.5 s; total spin-echo delay, 0.001 s; acquisition time, 2.72 s; selective irradiation of the water resonance signal, 4.712 ppm; and time-domain points, 64 K. Before Fourier transformation, the FIDs were multiplied by an exponential function equivalent to that of a 0.3 Hz line-broadening factor. The spectra were referenced to the TSP resonance at 0.0 ppm and manually corrected for the phase and baseline (MestReNova v. 11.0.3).

### Concentration counting and metabolites identification

All spectra were exported to Matlab (Matlab v. 8.3.0.532) for preprocessing. For ^1^H NMR signal identification and counting the metabolite concentration, Chenomx NMR Analysis Software (NMR suite v. 8.5, Chenomx Inc.) was used. The concentration of each compound was calculated by comparison to a reference signal—TSP with a known concentration of 0.3 mM. The metabolites for each strain were also checked in the KEGG database.

### Statistical data analysis

Statistical analysis was done in R software (version 4. 1.0) with gplots package (version 3.1.1). For all repetitions, distribution normality of data was tested with Shapiro–Wilk method, equity of variance was checked with Bartlett’s test. The repetitions were also checked with the Dixon test to see if one outlier value can be rejected. In comparison among bacteria strains and disintegration method, assumptions variance analysis with ANOVA method, followed by post hock analysis with HDS Tukey test were done for data samples fitting. For other data, Kruskal–Wallis alternative test was used, followed by the Wilcoxon signed-rank test for individual pairs of data sets with p-value correction according to the Benjamini–Hochberg procedure. Statistical significance was assumed at the p-value < 0.05. Additionally, the heatmap for each sample was generated. For this analysis, function 'heatmap.2' was used with default settings for hierarchical clustering.

### Multivariate data analysis

Multivariate data analysis was performed on a set of the assigned metabolites. To compare all samples—17 metabolites present in all samples were used (acetate, alanine, cholate, formate, glycine, isoleucine, lactate, lysine, leucine, methionine, NAD+, o-phosphocholine, phenylalanine, pyruvate, succinate, threonine, and valine). For comparison of gram-negative strains—18 metabolites common for this bacteria were used (uracil in addition to previously described metabolites). For comparison between gram-positive strains—26 metabolites common for this bacteria were used (compounds common for all strains and 4-aminobutyrate, aspartate, betaine, glutamate, histidine, inosine, sarcosine, tyramine, and tyrosine). The input for SIMCA-P (v 17.0, Umetrics, Umeå, Sweden) software was a transformed data matrix consisting of metabolite concentrations for each sample. The data sets were scaled using UV scaling before the chemometric analysis. For bacteria strains classification, principal component analysis (PCA) was carried out.

## Results

### Metabolites identification and concentration

On the obtained spectra, in total, 43 metabolites were identified. Not all metabolites were found in the spectrum of each strain. Eighteen common metabolites were identified for all 6 strains (acetate, alanine, cholate, formate, glycine, isoleucine, lactate, leucine, lysine, methionine, methanol, NAD+, o-phosphocholine, phenylalanine, pyruvate, succinate, threonine, valine). Some of the metabolites were identified for only one strain (in the *P. aeruginosa* spectrum:5-aminopentanoate and isocitrate; in the *E. feacalis* spectrum: asparagine and glutamine; in the *B. cereus* spectrum: uridine and β-alanine; in the *K. pneumoniae* spectrum: fumarate and nicotinate; in the *C. glutamicum* spectrum: trehalose).Other metabolites (4-aminobutyrate, adenine, adenosine, AMP, aspartate, betaine, glutamate, histidine, inosine, sarcosine, tyramine, oxypurinol, tyrosine, UDP-glucose, propylene glycol, uracil) have been identified in several strains.

**Figure 2:**
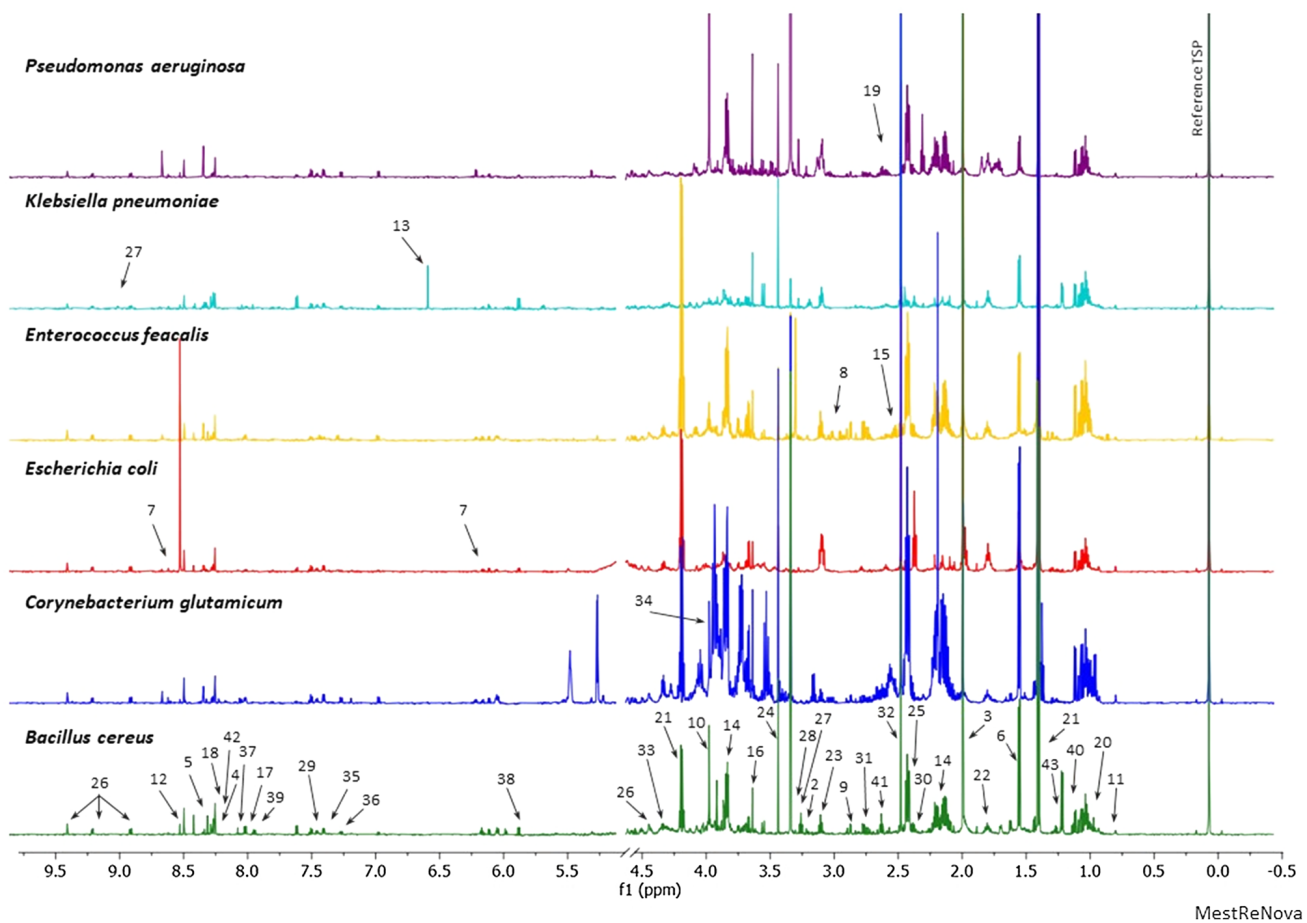
The representative 1D ^1^H NMR cpmgpr1d spectra obtained from different bacteria strains. (1: 5-aminopentanoate; 2: 4-aminobutyrate; 3: acetate; 4: adenine; 5: adenosine; 6: alanine; 7: AMP; 8: asparagine; 9: aspartate; 10: betaine; 11: cholate; 12: formate; 13: fumarate; 14: glutamate; 15: glutamine; 16: glycine; 17: histidine; 18: inosine; 19: isocitrate; 20: isoleucine; 21: lactate; 22: leucine; 23: lysine; 24: methanol; 25: methionine; 26: NAD+; 27: nicotinate; 28: O-phosphocholine; 29: phenylalanine; 30: pyruvate; 31: sarcosine; 32: succinate; 33: threonine; 34: trehalose; 35: tyramine; 36: tyrosine; 37: UDP-glucose; 38: uracil; 39: uridine; 40: valine; 41: β-alanine; 42: oxypurinol; 43: propylene glycol).

Representative ^1^H NMR spectra obtained from different bacteria strains with marked identified metabolites are presented below (Fig. 2). A more detailed representation of the identified peaks for each of the tested strains is available in the supplementary materials (Figures S2, S3), where information about the chemical shift for each metabolite are deposited in Table S1.

In *E. coli* strain 23 metabolites were identified, while in *B. cereus*—34 and in *C. glutamicum*—31. The number of identified metabolites in *E. feacalis* and *K. pneumoniae *was 30. In *P. aeruginosa* 28 metabolites were identified. For each sample, the concentration of the metabolites was calculated. The cell disruption method did not affect the number of identified metabolites. The data about average concentration with the standard deviation are presented in the supplementary materials (Tables S2, S3).

### Methods comparison—statistical analysis

**Figure 3:**
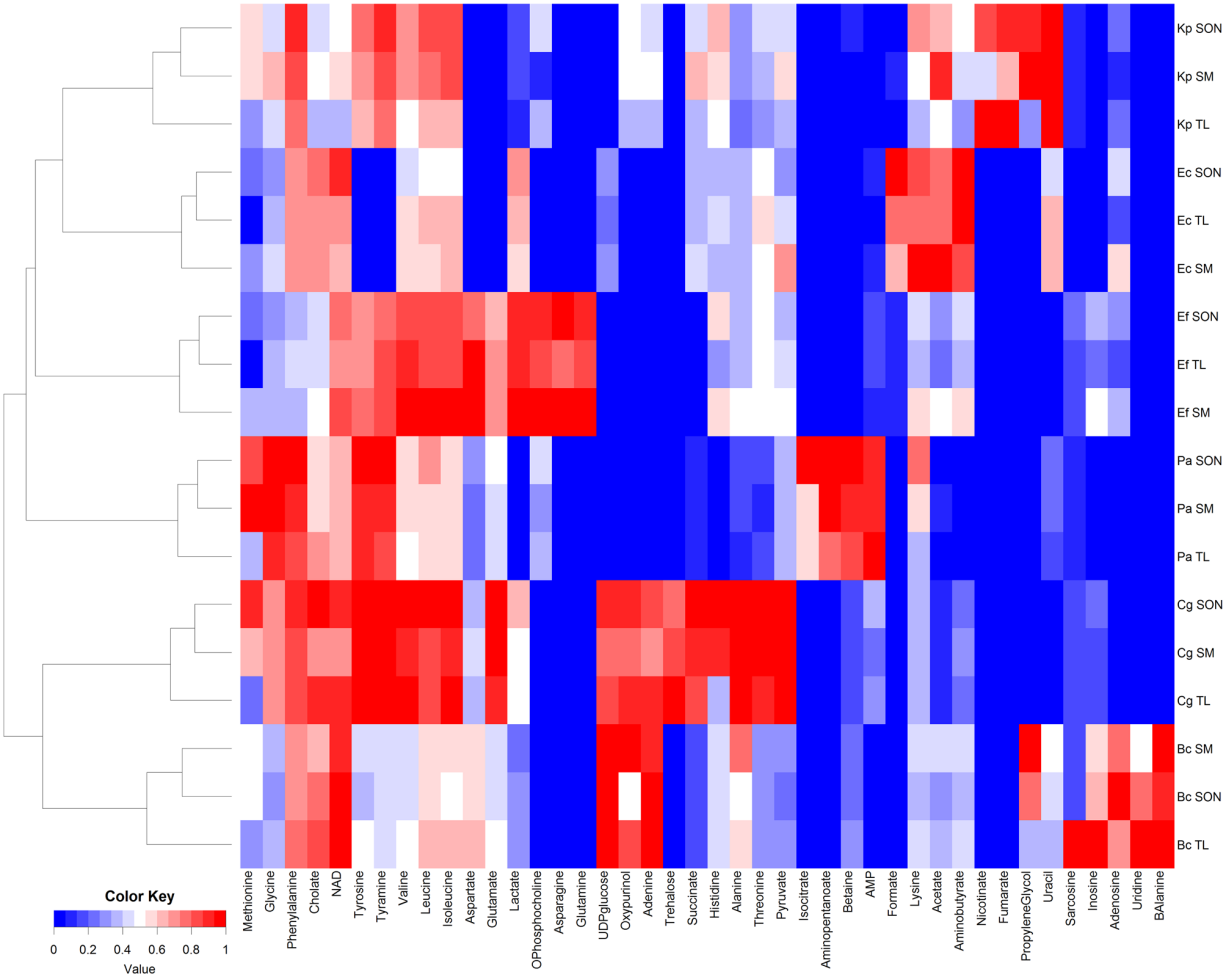
Heat-map comparing the average concentration of metabolites. The heat map was generated by hierarchical clustering analysis (HCA) of all identified metabolites. Red color represents high metabolite concentration and intense blue color represents very low metabolite concentration or metabolite absence (SM—sand mill; TL—tissue lyser; SON—sonication; BC—*B. cereus*; CG—*C. glutamicum*; EF—*E. feacalis*; EC—*E. coli*; KP—*K. pneumoniae*; PA—*P. aeruginosa*).

The changes in the metabolite concentrations, which depend on the disintegration methods are present on the heatmap (Fig. 3). Hierarchical clustering of average metabolite concentrations resulted in the grouping of samples from individual species in separate clusters. Furthermore, this analysis revealed that for *K. pneumoniae*, *E. coli*, and *E. feacalis* the most differentiating method is sand mill. The tissue lyser was the most distinguishing method in *P. aeruginosa* and *C. glutamicum* strain, while for *B. cereus*—sonication was different from the other disintegration processes.

The results obtained on the heat-map (Fig. 3) should be analyzed together with statistical analysis results (Table 2). This analysis was performed to find out the differences between the three disintegration methods for each strain.

Among all metabolites identified for *B. cereus*, the statistically significant differences between disintegration methods were obtained for alanine, formate, methionine, o-phosphocholine, propylene glycol, sarcosine, and uridine. Comparing the metabolite concentrations obtained by three different methods for *Bacillus cereus* samples showed that the highest average concentrations of 25 metabolites were found after using the tissue lyser instrument, accounting almost 74% of all identified metabolites in this strain. However, only in the case of sarcosine, the difference was statistically significant. Sonication of *B. cereus* samples allowed us to obtain the highest concentration for two metabolites and was statistically significant only for formate. The sand mill gave the highest yield of extraction for other metabolites, and the difference was statistically significant for alanine and methionine.

In gram-positive species, *Enterococcus faecalis*, statistically significant differences between disintegration methods were found for nine metabolites—4-aminobutyrate, acetate, adenine, alanine, lysine, methionine, pyruvate, and sarcosine. Disintegration using a sand mill gave the highest concentrations for twenty-six compounds, accounting almost 87% of all identified metabolites. Eight metabolites were statistically significant. Sonication gave the highest concentration in the case of three metabolites, but only sarcosine turned out to be significant. Tissue lyser use gave the highest amount of phenylalanine.

The analysis of the third gram-positive strain, *Corynebacterium glutamicum*, showed that the statistically significant differences between disintegration methods were obtained for nine metabolites—adenine, cholate, formate, histidine inosine, lactate, methionine, NAD+, and oxypurinol. Sonication gave the highest concentration of most of the identified metabolites—twenty, which consist more than 64% of all identified metabolites, but the statistical importance was obtained for six compounds. The tissue lyser yielded the highest concentration for six metabolites, among which three were statistically different. Additionally, five metabolites were found at the highest level when the sand mill was used, but no one was statistically important.

When we compared the metabolite concentrations in *Escherichia coli* samples, the highest concentrations of eleven metabolites were obtained after sand milling. Among these compounds, seven were statistically significant—acetate, adenosine, alanine, AMP, glycine, lysine, and pyruvate. Sonication and tissue lyser of these samples allowed us to obtain the highest concentrations for six metabolites. For sonication, four metabolites were statistically significant (cholate, lactate, methionine, and NAD+), when tissue lyser gives only two differentiating metabolites—formate and o-phosphocholine.

In the case of a different gram-negative strain, *Pseudomonas aeruginosa*, the statistically significant differences between disintegration methods were obtained for nine—5-aminopentanoate, acetate, alanine, cholate, formate, isocitrate, lysine, methionine, and o-phosphocholine. For almost all identified metabolites, disintegration using sonication yielded the highest concentrations. It is 19 metabolites, which consist almost 68% of all identified metabolites in this strain, among which seven were statistically significant. The tissue lyser yielded the highest concentration for three metabolites, but only cholate was statistically significant. Sand milling yielded the highest amounts of six metabolites, but the result was statistically significant for acetate.

Analysis of the third gram-negative strain, *Klebsiella pneumoniae*, allowed to obtained seven statistically important metabolites—acetate, alanine, cholate, glycine, lysine, methionine, and NAD+. Sonication yielded the highest concentrations of sixteen identified metabolites, of which three were statistically important. The tissue lyser enabled us to obtain the highest concentrations of three metabolites, no one of them was significant. When the sand mill was used, the highest concentrations of eleven metabolites were found. However, only four of them were statistically important.

To investigate if all disintegration methods give the same information about the average concentration relation and statistical comparison among gram-positive and gram-negative strains, additional analyses were performed.

In the group of gram-negative strains, the all average level ratio of common metabolites was almost the same in acetate, alanine, cholate, formate, glycine, lactate, leucine, lysine, methionine, NAD+, o-phosphocholine, phenylalanine, succinate, threonine, and uracil. Three metabolites had different relations of the concentration average—isoleucine, pyruvate, valine. Statistical analysis performed on one disintegration method allowed to obtained many statistical importance differences between strains, but the results are not similar in each disintegration method. Detailed information about these analyses is available in supplementary materials (Table S4).

Analogical analyses were performed for gram-positive strains. In this case, the average concentration relation wasn’t the same in almost half of the common metabolites. Also for statistical analyses performed on one disintegration method, the statistical important metabolites are not similar in each disintegration method. Detailed information about analyses performed in gram-positive strains is available in supplementary materials (Table S5).

### Multivariate data analysis

**Figure 4:**
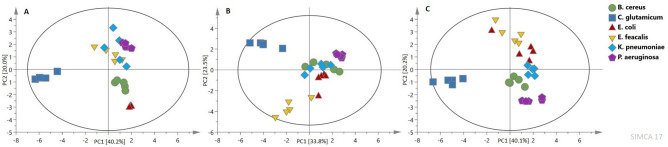
PCA score plots for each one disintegration method for all strains: (**A**) sonication, (**B**) sand mill, (**C**) tissue lyser.

Multivariate data analyses were performed to compare disintegration methods for different bacteria strains. PCA score plots show how disintegration methods influence multivariate data analysis. This unsupervised comparison allowed to obtain grouping of samples according to the type of microorganism for each disintegration method. It is worth to mentioning that this type of chemometric analysis distinctively reflects the similarities and differences of the cell disruption method on studied bacterial strands. The PCA model for the samples subjected to sonication was prepared based on seven PCs with the total variance in the data equal 0.982. The sand mill model consists of six PC’s and with an R2X value of 0.955, while the tissue lyser model consists of two PC’s and with an R2X value equal 0.603 (Fig. 4).

The results showed, that each of the used disintegration methods can be applied in metabolomics studies, but for the data preparation only one of them should be chosen.

Besides comparison on all strains, the analyzes were conducted among the limited data. One of them is the comparisons made separately for the gram-positive strains and gram-negative strains. In this case, we can observe the clear separation of each strain comparing samples together and separately for each disintegration method, while PCA analysis is performed. The models and their parameters are available in supplementary materials (Figure S4).

**Figure 5:**
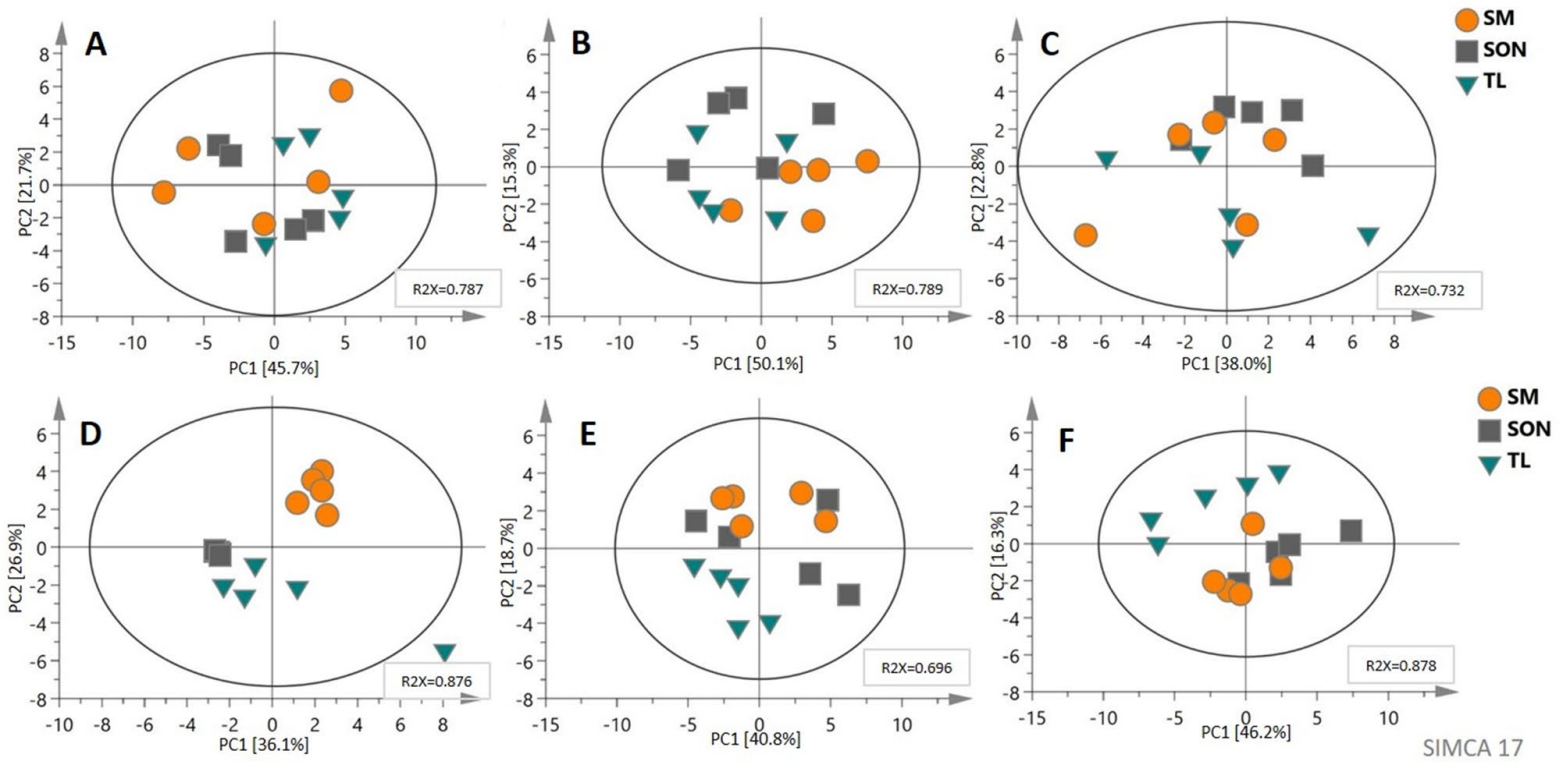
PCA models for separate analysis of each single bacterium strain. (**A**) *B. cereus*; (**B**) *E. feacalis*; (**C**) *C. glutamicum*; (**D**) *E. coli*; (**E**) *K. pneumoniae*; (**F**) *P. aeruginosa* (SM—sand mill; SON—sonication; TL—tissue lyser).

Additionally, multivariate analyses for each strain separately were prepared to obtained information about samples grouping for different disintegration methods. These results showed that among all strains, we can observe distinguished groups for the disintegration method only in *E. coli*, while for some other strains the clustering trends are outlined. The remaining PCA analyzes performed for the single bacterium illustrate that samples prepared with different disintegration methods are similar or overlap (Fig. 5).

## Discussion

The adequately prepared protocols are essential in research studies. In metabolomics, the fundamental issue is the sample preparation, which allows performing reliable analyzes.

Many protocols are based on cell disintegration—this step is especially important when intracellular metabolites analyses are performed. To compare the differences between the disruption method, six bacteria strains were used in the study—three gram-positive strains and three gram-negative strains. LB medium used for bacterial cultivation was additionally enriched with 0.5% glucose to obtain better biomass growth, which allowed to obtain enough material for analyses. These changes were necessary because some strains are growing very poorly, and the typical LB medium does not allow to obtain the required weight of the lyophilisate. Additionally, for each strain, growth curves were prepared. During bacterial growth, changes in bacterial metabolism occurred, and these results allowed to determine when all exanimated bacterial strains were in the logarithmic growth phase. In this phase, the division of bacteria is continuous at a constant rate, and the number of cells increases exponentially. Furthermore, the bacterial population in this phase is nearly uniform in terms of their number, chemical composition, other physiological properties of the cell, and metabolic activity^35,36^.

The extraction procedure was performed on lyophilized samples, giving us the possibility to accurately determine each sample's biomass. The methodological advantages of this bacterial preparation form for metabolomic applications are the possibility of longer storage/transport, ease of measuring the same number of cells (mass provides sufficient information), and reduction of the extraction scale (this involves a large number of samples that are compared in metabolomics)^37,38^.

In metabolomics studies, many different solvents and procedures are using for sample preparation. We decided to use for extraction water and methanol. The use of polar solvents allowed us to obtain a wide range of compounds in the samples. Additionally, from the practical preparation way, this method seems to be for us the most proper.

The conducted experiment confirmed that each of the disintegration methods allows obtaining similar metabolites in samples of the same strains. The obtained differences are likely due to the different cell wall structures. As described above, the difference in the cell wall structure between gram-negative and gram-positive bacteria is obvious and influences mechanical resistance^26^. If we compare these two groups of bacteria, gram-negative bacteria have a relatively thin layer of peptidoglycan, and the cell wall can be destroyed by the action of pressure^30,31^.

It is hard to define if a given disintegration method the best for a specific bacteria strain. Among all investigated strains, the identified metabolites concentrations were different for different disruption methods. In all cases, most metabolites' level was the highest for one of the disruption methods, but there is no strain in which we obtained the highest concentration for one disruption method. For example, in *E. feacalis*, almost 87% of metabolites had the highest concentration when the sand mill was used, but some had the highest concentration after sonication or tissue lyser use. We can observe in *B. cereus* in the analogical situation that almost 74% of the highest concentrations were obtained with tissue lyser (Tables S2 and S3). These results can be helpful in targeted analysis, where a specific group of compounds or individual metabolites should be studied. If one specific compound should be investigated, it is worth checking if some commonly used disintegration methods can yield the highest concentration. In many cases, the differences between obtained concentrations are not significant, but these differences are essential for some compounds and can influence the analysis. This fact clearly showed the importance, in metabolomics studies, of correct and consistent sample preparation. During data interpretation, we must remember about a limited number of biological repetitions. It is possible that experimenting with more repetitions for each sample would give a more accurate result.

Multivariate data analyses are typical for metabolomics studies. The untargeted method (PCA) performed on all microorganism samples, allowed us to obtain the natural strains grouping (Fig. 3). The analogical results were obtained, where the disintegration methods were compared separately. In all cases, the distinction of bacteria strains is possible (Fig. 4). These results and many published results indicate that NMR can be useful for identifying and distinguishing bacterial strains; however, MS is currently more widely used^2,10,39^. This finding indicates that each bacterial strain has its metabolomic qualitative profile regardless of the disruption method (only the metabolite concentrations change).

The same results—clearly natural grouping of samples for each strain –can be observed when gram-positive and gram-negative strains are compared (regardless of the disintegration method chosen) (Figure S4). Besides the comparison of different bacteria strains depending on the disruption methods, the PCA models comparing the disintegration methods for each strain were performed (Fig. 5). Among all comparisons only in *E. coli* showed a clear grouping of samples depending on the disruption methods, where the most metabolites showed statistical importance. These results showed that all from the used disruption method can be useful for untargeted metabolomics analysis. Additionally, these results can allow the selection of the best method of sample preparation to analyze specific compounds, which are important in targeted metabolomics.

## Conclusions

The performed experiments provide results that can be used in different areas of microbial metabolomics experiments and demonstrated the importance of accurately chosen sample preparing protocols. Our findings confirms that the disintegration method impacts the extraction quality (thus, comparing samples prepared by different methods may lead to false results) and should be selected for specific bacterial microorganisms. It is worth to mention that the disintegration methods do not influence the qualitative profile of intracellular microbial metabolites changing only their concentration.

## Supplementary Information

Supplementary Information.
