# Supplementary material for: Comparison of bacteria disintegration methods and their influence on data analysis in metabolomics
Source: Sci Rep. 2021 Oct 21;11:20859. doi: 10.1038/s41598-021-99873-x (PMC8531443; doi:10.1038/s41598-021-99873-x)
Supplement: Supplementary file 1 — Supplementary Information. [file 41598_2021_99873_MOESM1_ESM.pdf]

Comparison of bacteria disintegration methods and their influence on data analysis in metabolomics

Karolina Anna Mielko[1], Sławomir Jabłoński[2], Marcin Łukaszewicz[2], Piotr Młynarz[1]*

[1] *Department of Biochemistry, Molecular Biology and Biotechnology, Faculty of Chemistry, Wroclaw University of Science and Technology;*
[2] *Biotransformation Department, Faculty of Biotechnology, University of Wroclaw*

*corresponding author: piotr.mlynarz@pwr.edu.pl

SUPPLEMENTARY MATERIALS

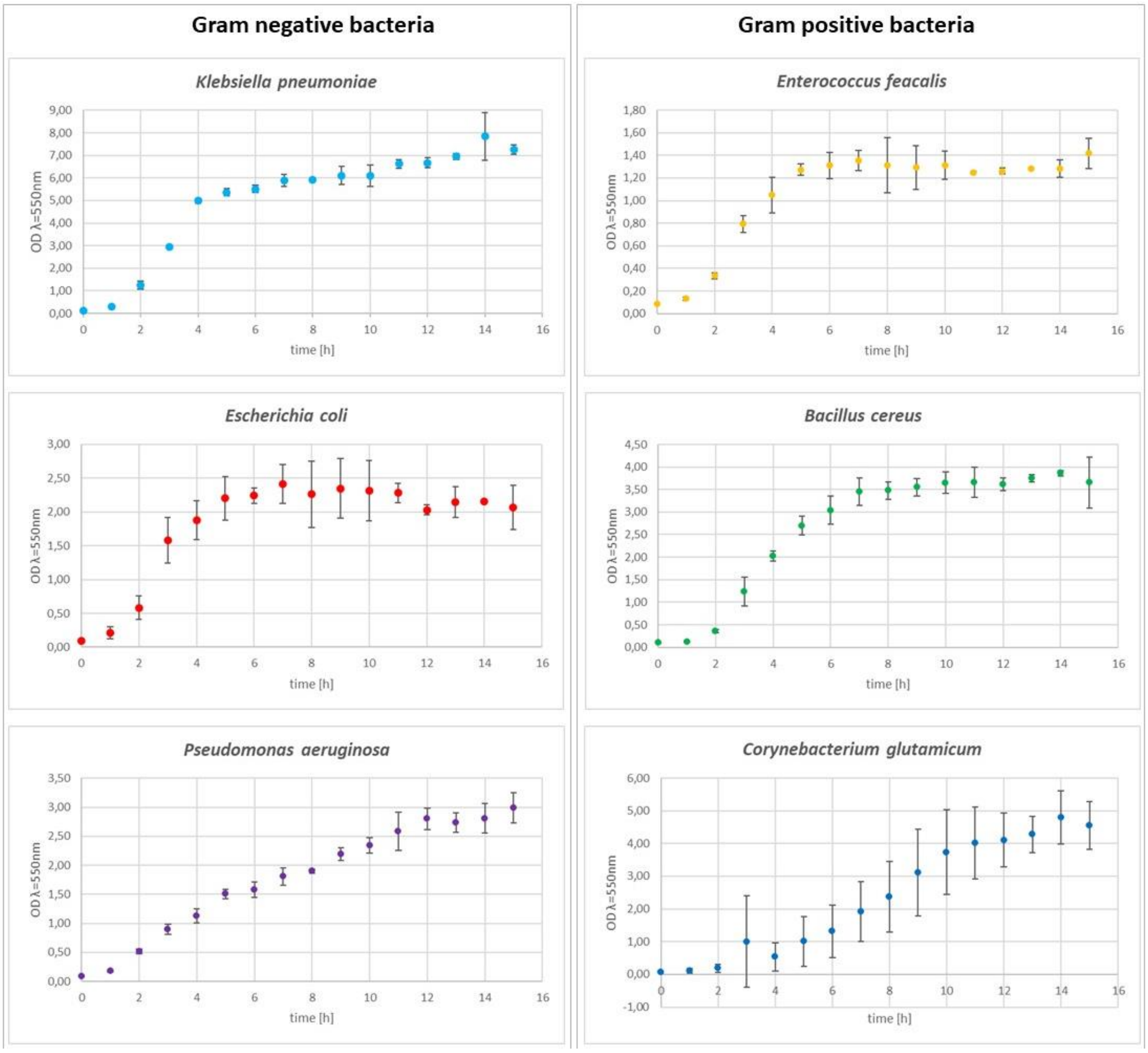

Figure S1. Growth curves of bacterial strains.

Table S1. ^{1}H NMR signal assignments.

L.p.	Compound	KEGG Identification number	Chemicalshift [ppm]
**1.**	***5-aminopentanoate***	C00431	3.0; 2.2; 1.6
**2.**	***4-Aminobutyrate***	C15987	2.3
**3.**	***Acetate***	C00036	1.9
**4.**	***Adenine***	C00147	8.2
**5.**	***Adenosine***	C00212	8.3; 6.1
**6.**	***Alanine***	C00041	3.8; 1.5
**7.**	***AMP***	C00360	8.6; 8.3; 6.1
**8.**	***Asparagine***	C00152	2.9
**9.**	***Aspartate***	C00049	2.8
**10.**	***Betaine***	C00719	3.9; 3.3
**11.**	***Cholate***	C02528	0.7
**12.**	***Formate***	C00058	8.4
**13.**	***Fumarate***	C00122	6.5
**14.**	***Glutamate***	C00025	3.7; 2.4;2.3; 2.1; 2.0
**15.**	***Glutamine***	C00064	2.5; 2.4
**16.**	***Glycine***	C00037	3.6
**17.**	***Histidine***	C00135	7.1
**18.**	***Inosine***	C00294	8.2
**19.**	***Isocitrate***	C00311	2.6; 2.5
**20.**	***Isoleucine***	C00407	0.9
**21.**	***Lactate***	C00256	4.1; 1.3
**22.**	***Leucine***	C00123	1.0
**23.**	***Lysine***	C00047	1.7
**24.**	***Methanol***	C00132	3.4
**25.**	***Methionine***	C00073	2.6; 2.1
**26.**	***NAD+***	C00003	9.3; 9.1; 8.8; 8.4; 4.4
**27.**	***Nicotinate***	C00253	8.9
**28.**	***O-Phosphocholine***	C04230	3.2
**29.**	***Phenylalanine***	C02265	7.4; 7.3
**30.**	***Pyruvate***	C00022	2.4
**31.**	***Sarcosine***	C00213	2.7
**32.**	***Succinate***	C00042	2.4
**33.**	***Threonine***	C00188	3.6
**34.**	***Trehalose***	C01083	5.2; 3.8; 3.4
**35.**	***Tyramine***	C00483	7.2; 6.9
**36.**	***Tyrosine***	C00082	7.2; 6.9
**37.**	***UDP-glucose***	C00029	7.9; 6.0
**38.**	***Uracil***	C00106	7.5; 5.8
**39.**	***Uridine***	C00299	7.9; 5.9
**40.**	***Valine***	C00183	3.6; 1.0
**41.**	***β-Alanine***	C00099	3.2; 2.5
**42.**	***Oxypurinol***	C07599	8.2
**43.**	***Propylene glycol***	C00583	1.1

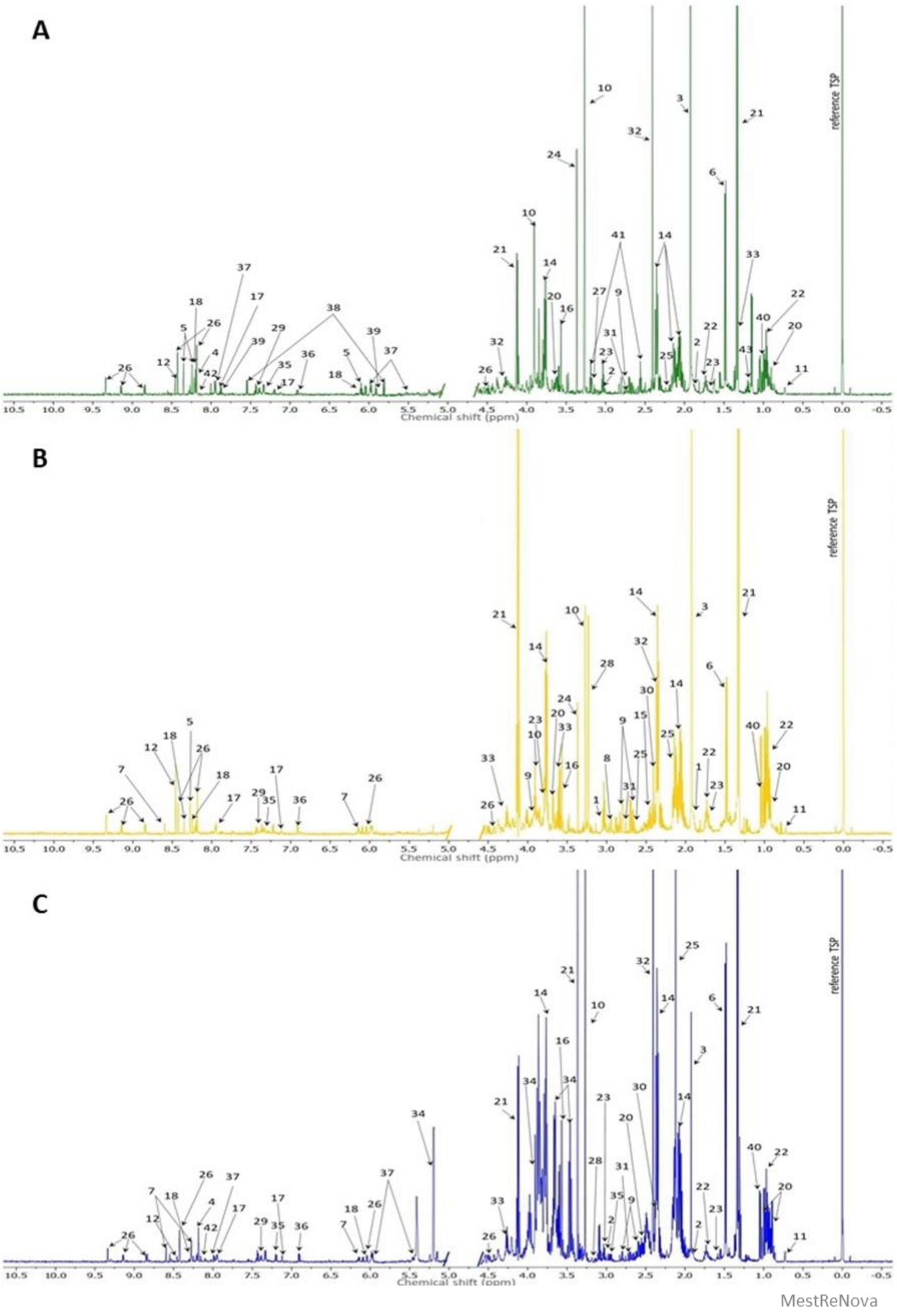

Figure S2. The representative 1D ^{1}H NMR spectra of gram-positive bacteria samples. A – *Bacillus cereus,* B – *Enterococcus feacalis,* C – *Corynebaterium glutamicum*

(1 – 5-aminopentanoate; 2 – 4-aminobutyrate; 3 – acetate; 4 – adenine; 5 – adenosine; 6 – alanine; 7 – AMP; 8 – asparagine; 9 – aspartate; 10 – betaine; 11 – cholate; 12 – formate; 13 – fumarate; 14 – glutamate; 15 – glutamine; 16 – glycine; 17 – histidine; 18 – inosine; 19 – isocitrate; 20 – isoleucine; 21 – lactate; 22 – leucine; 23 – lysine; 24 – methanol; 25 – methionine; 26 – NAD+; 27 – nicotinate; 28 – O-phosphocholine; 29 – phenylalanine; 30 – pyruvate; 31 – sarcosine; 32 – succinate; 33 – threonine; 34 – trehalose; 35 – tyramine; 36 – tyrosine; 37 – UDP-glucose; 38 – uracil; 39 – uridine; 40 – valine; 41 – β-alanine; 42 – oxypurinol; 43 – propylene glycol)

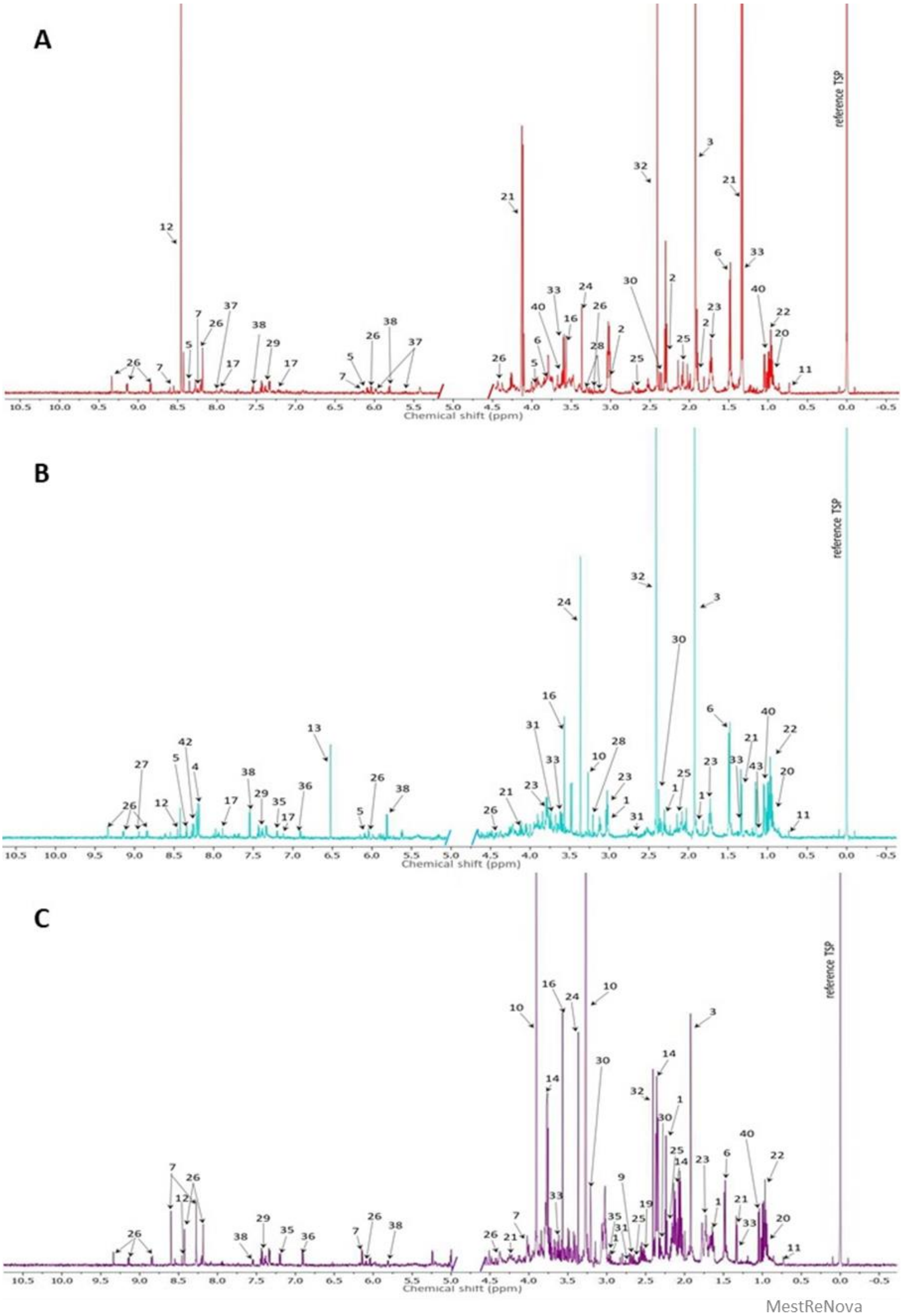

Figure S3. The representative 1D ^{1}H NMR spectra of gram-negative bacteria samples. A – *Escherichia coli*, B– *Klebsiella pneumoniae*, C – *Pseudomonas aeruginosa*

(1 – 5-aminopentanoate; 2 – 4-aminobutyrate; 3 – acetate; 4 – adenine; 5 – adenosine; 6 – alanine; 7 – AMP; 8 – asparagine; 9 – aspartate; 10 – betaine; 11 – cholate; 12 – formate; 13 – fumarate; 14 – glutamate; 15 – glutamine; 16 – glycine; 17 – histidine; 18 – inosine; 19 – isocitrate; 20 – isoleucine; 21 – lactate; 22 – leucine; 23 – lysine; 24 – methanol; 25 – methionine; 26 – NAD+; 27 – nicotinate; 28 – O-phosphocholine; 29 – phenylalanine; 30 – pyruvate; 31 – sarcosine; 32 – succinate; 33 – threonine; 34 – trehalose; 35 – tyramine; 36 – tyrosine; 37 – UDP-glucose; 38 – uracil; 39 – uridine; 40 – valine; 41 – β-alanine; 42 – oxypurinol; 43 – propylene glycol)

Table S2. Metabolites concentration with standard deviation [mM] for gram-negative strains (SM – sand mill; TL – tissue lyser; SON – sonication).

Metabolite	*E. coli*			*K. pneumoniae*			*P. aeruginosa*		
	SM	TL	SON	SM	TL	SON	SM	TL	SON
5-aminopentanoate	-	-	-	-	-	-	0.197±0.023	0.159±0.019	0.207±0.033
4-Aminobutyrate	0.259±0.046	0.306±0.123	0.290±0.006	0.124±0.054	0.084±0.012	0.163±0.031	-	-	-
Acetate	2.386±0.175	1.801±0.175	1.818±0.026	2.216±0.132	1.252±0.378	1.472±0.265	0.188±0.017	0.111±0.019	0.151±0.017
Adenine	-	-	-	0.088±0.024	0.063±0.016	0.079±0.020	-	-	-
Adenosine	0.031±0.003	0.008±0.002	0.022±0.000	0.007±0.001	0.012±0.006	0.013±0.002	-	-	-
Alanine	0.166±0.004	0.145±0.017	0.148±0.003	0.128±0.021	0.106±0.005	0.138±0.025	0.068±0.006	0.055±0.008	0.081±0.009
AMP	0.012±0.002	0.004±0.003	0.011±0.000	-	-	-	0.085±0.005	0.091±0.015	0.082±0.009
Aspartate	-	-	-	-	-	-	0.056±0.009	0.05±0.0130	0.060±0.010
Betaine	-	-	-	0.063±0.078	0.068±0.076	0.088±0.102	0.942±0.022	0.893±0.138	1.035±0.070
Cholate	0.010±0.000	0.010±0.001	0.011±0.000	0.007±0.001	0.005±0.001	0.006±0.001	0.008±0.001	0.010±0.001	0.008±0.000
Formate	0.739±0.063	0.862±0.247	1.124±0.018	0.107±0.141	0.034±0.034	0.026±0.017	0.019±0.001	0.011±0.001	0.024±0.004
Fumarate	-	-	-	0.015±0.008	0.024±0.024	0.022±0.029	-	-	-
Glutamate	-	-	-	-	-	-	0.812±0.061	0.788±0.085	0.859±0.048
Glycine	0.081±0.003	0.072±0.017	0.054±0.001	0.120±0.021	0.084±0.004	0.088±0.022	0.196±0.017	0.181±0.022	0.192±0.005
Histidine	0.012±0.002	0.014±0.006	0.013±0.000	0.017±0.001	0.017±0.002	0.021±0.004	-	-	-
Isocitrate	-	-	-	-	-	-	0.097±0.002	0.095±0.028	0.162±0.018
Isoleucine	0.039±0.002	0.036±0.012	0.029±0.000	0.049±0.006	0.039±0.004	0.048±0.010	0.033±0.002	0.033±0.007	0.035±0.005
Lactate	1.047±0.137	1.250±0.255	1.329±0.013	0.275±0.235	0.247±0.170	0.350±0.267	0.036±0.007	0.036±0.009	0.044±0.010
Leucine	0.076±0.005	0.082±0.024	0.067±0.003	0.099±0.017	0.086±0.010	0.106±0.026	0.077±0.006	0.070±0.015	0.087±0.008
Lysine	0.377±0.024	0.287±0.053	0.312±0.007	0.196±0.070	0.135±0.018	0.251±0.049	0.215±0.010	0.143±0.034	0.280±0.021
Methionine	0.020±0.003	0.020±0.001	0.020±0.000	0.025±0.011	0.023±0.007	0.031±0.015	0.036±0.003	0.033±0.003	0.041±0.002
NAD+	0.062±0.006	0.068±0.009	0.087±0.001	0.055±0.012	0.037±0.010	0.050±0.004	0.062±0.002	0.065±0.007	0.065±0.004
Nicotinate	-	-	-	0.011±0.003	0.026±0.017	0.022±0.017	-	-	-
O-Phosphocholine	0.002±0.000	0.0020	0.002±0.000	0.005±0.003	0.017±0.013	0.019±0.016	0.013±0.003	0.017±0.003	0.020±0.003
Oxypurinol	-	-	-	0.586±0.197	0.475±0.122	0.640±0.235	-	-	-
Phenylalanine	0.040±0.008	0.0410±0.006	0.042±0.001	0.050±0.008	0.044±0.007	0.054±0.017	0.053±0.005	0.051±0.010	0.060±0.010
Propyleneglycol	-	-	-	0.089±0.043	0.029±0.020	0.081±0.067	-	-	-
Pyruvate	0.042±0.007	0.026±0.001	0.018±0.000	0.034±0.015	0.023±0.001	0.026±0.010	0.020±0.001	0.019±0.002	0.020±0.001
Sarcosine	-	-	-	0.003±0.001	0.002±0.000	0.003±0.001	0.002±0.000	0.002±0.001	0.002±0.000
Succinate	0.266±0.008	0.232±0.048	0.215±0.004	0.388±0.224	0.247±0.187	0.287±0.198	0.065±0.008	0.058±0.006	0.062±0.007
Threonine	0.189±0.003	0.205±0.019	0.193±0.003	0.139±0.060	0.120±0.038	0.152±0.067	0.041±0.008	0.054±0.011	0.055±0.014
Tyramine	-	-	-	0.024±0.004	0.022±0.002	0.027±0.007	0.026±0.004	0.024±0.003	0.028±0.003
Tyrosine	-	-	-	0.023±0.004	0.020±0.002	0.025±0.007	0.027±0.002	0.027±0.004	0.029±0.003
UDP-glucose	0.015±0.002	0.013±0.001	0.017±0.000	-	-	-	-	-	-
Uracil	0.040±0.002	0.040±0.013	0.029±0.001	0.061±0.021	0.063±0.018	0.06±0.019	0.016±0.004	0.011±0.003	0.013±0.003
Valine	0.065±0.007	0.064±0.019	0.054±0.001	0.080±0.010	0.062±0.002	0.08±0.015	0.064±0.004	0.061±0.007	0.068±0.006

*the concentrations calculated for 4 biological replicates are marked in gray (the Dixon test was used to reject mismatched samples)

Table S3. Metabolites concentration with standard deviation [mM] for gram-positive strains (SM – sand mill; TL – tissue lyser; SON – sonication).

Metabolite	*B. cereus*			*E. feacalis*			*C. glutamicum*		
	SM	TL	SON	SM	TL	SON	SM	TL	SON
4-Aminobutyrate	0.130±0.028	0.125±0.007	0.121±0.019	0.180±0.031	0.117±0.021	0.124±0.026	0.092±0.014	0.080±0.004	0.076±0.004
Acetate	1.086±0.343	0.855±0.252	0.728±0.277	1.271±0.218	0.626±0.120	0.735±0.266	0.238±0.038	0.214±0.062	0.185±0.044
Adenine	0.152±0.020	0.170±0.009	0.164±0.003	-	-	-	0.119±0.012	0.150±0.017	0.145±0.011
Adenosine	0.041±0.013	0.038±0.011	0.054±0.003	0.019±0.003	0.008±0.006	0.015±0.004	-	-	-
Alanine	0.321±0.048	0.252±0.038	0.219±0.023	0.203±0.022	0.148±0.017	0.148±0.021	0.406±0.040	0.421±0.057	0.424±0.039
AMP	-	-	-	0.008±0.003	0.012±0.002	0.015±0.005	0.022±0.009	0.028±0.010	0.034±0.004
Asparagine	-	-	-	0.073±0.017	0.054±0.008	0.070±0.015	-	-	-
Aspartate	0.117±0.036	0.137±0.017	0.125±0.032	0.213±0.020	0.202±0.039	0.168±0.033	0.086±0.011	0.079±0.018	0.076±0.018
Betaine	0.252±0.092	0.281±0.080	0.245±0.071	0.049±0.020	0.041±0.010	0.047±0.014	0.155±0.010	0.154±0.010	0.164±0.020
Cholate	0.009±0.002	0.012±0.002	0.011±0.002	0.007±0.000	0.006±0.001	0.006±0.001	0.010±0.001	0.013±0.001	0.014±0.000
Formate	0.023±0.007	0.014±0.006	0.029±0.010	0.131±0.009	0.093±0.036	0.134±0.048	0.011±0.001	0.008±0.001	0.016±0.001
Glutamate	0.779±0.179	0.829±0.069	0.776±0.102	1.275±0.147	1.229±0.246	1.085±0.089	1.766±0.311	1.610±0.152	1.776±0.178
Glutamine	-	-	-	0.084±0.024	0.073±0.011	0.077±0.027	-	-	-
Glycine	0.069±0.020	0.073±0.008	0.060±0.003	0.075±0.000	0.066±0.008	0.063±0.013	0.132±0.012	0.137±0.021	0.141±0.010
Histidine	0.014±0.005	0.014±0.002	0.013±0.002	0.018±0.005	0.010±0.007	0.018±0.001	0.029±0.003	0.012±0.006	0.032±0.004
Inosine	0.023±0.008	0.042±0.012	0.027±0.013	0.021±0.004	0.009±0.006	0.017±0.006	0.006±0.001	0.006±0.002	0.010±0.000
Isoleucine	0.035±0.008	0.039±0.004	0.032±0.004	0.059±0.010	0.050±0.008	0.048±0.012	0.055±0.007	0.058±0.006	0.059±0.004
Lactate	0.500±0.0350	0.611±0.158	0.550±0.089	1.890±0.324	1.704±0.250	1.711±0.299	1.008±0.170	0.940±0.101	1.208±0.042
Leucine	0.076±0.021	0.085±0.012	0.076±0.009	0.129±0.005	0.105±0.016	0.107±0.029	0.109±0.009	0.111±0.015	0.122±0.005
Lysine	0.153±0.035	0.154±0.011	0.142±0.024	0.222±0.012	0.149±0.028	0.153±0.037	0.134±0.013	0.133±0.014	0.135±0.004
Methionine	0.029±0.006	0.036±0.004	0.031±0.005	0.060±0.007	0.052±0.006	0.046±0.007	0.444±0.033	0.420±0.009	0.445±0.007
NAD+	0.089±0.012	0.099±0.004	0.094±0.004	0.081±0.007	0.071±0.007	0.073±0.014	0.071±0.008	0.088±0.012	0.088±0.007
O-Phosphocholine	0.002±0.001	0.003±0.000	0.002±0.001	0.046±0.017	0.039±0.008	0.041±0.011	0.002±0.000	0.002±0.000	0.002±0.000
Oxypurinol	1.202±0.100	0.992±0.383	0.599±0.393	-	-	-	0.892±0.096	1.121±0.125	1.104±0.091
Phenylalanine	0.04±0.0100	0.047±0.007	0.041±0.006	0.024±0.006	0.025±0.006	0.023±0.004	0.050±0.005	0.052±0.007	0.052±0.004
Propyleneglycol	0.093±0.033	0.034±0.009	0.070±0.024	-	-	-	-	-	-
Pyruvate	0.016±0.004	0.018±0.002	0.015±0.002	0.029±0.003	0.025±0.004	0.023±0.000	0.057±0.007	0.054±0.004	0.058±0.005
Sarcosine	0.005±0.001	0.025±0.008	0.003±0.001	0.004±0.001	0.003±0.001	0.006±0.001	0.004±0.001	0.004±0.000	0.004±0.001
Succinate	0.095±0.033	0.113±0.034	0.097±0.038	0.030±0.007	0.026±0.004	0.027±0.002	0.585±0.151	0.531±0.109	0.633±0.081
Threonine	0.112±0.042	0.116±0.022	0.107±0.028	0.177±0.024	0.177±0.031	0.171±0.018	0.348±0.019	0.336±0.035	0.362±0.038
Trehalose	-	-	-	-	-	-	0.452±0.124	0.525±0.180	0.398±0.033
Tyramine	0.012±0.003	0.013±0.003	0.013±0.004	0.025±0.004	0.024±0.004	0.022±0.003	0.029±0.001	0.029±0.003	0.028±0.002
Tyrosine	0.013±0.004	0.015±0.002	0.012±0.003	0.025±0.004	0.023±0.003	0.022±0.003	0.031±0.002	0.031±0.004	0.031±0.002
UDP-glucose	0.051±0.009	0.054±0.007	0.050±0.004	-	-	-	0.041±0.012	0.046±0.008	0.048±0.009
Uracil	0.033±0.013	0.024±0.010	0.026±0.015	-	-	-	-	-	-
Uridine	0.015±0.004	0.030±0.006	0.023±0.009	-	-	-	-	-	-
Valine	0.055±0.012	0.060±0.007	0.049±0.006	0.117±0.016	0.103±0.018	0.099±0.018	0.108±0.011	0.110±0.007	0.115±0.009
β-Alanine	0.045±0.013	0.042±0.016	0.041±0.015	-	-	-	-	-	-

*the concentrations calculated for 4 biological replicates are marked in gray (the Dixon test was used to reject mismatched samples)

Table S4. Variation of metabolite concentration between gram-negative bacteria within disruption method. (Ec – *E. coli*; Kp – *K. pneumoniae*; Pa – *P. aeruginosa*)

Metabolite	Statistical comparison						Average concentration relation		
	Sonication		Sand mill		Tissue Lyser		Sonication	Sand mill	Tissue Lyser
	p-value	Interpretation	p-value	Interpretation	p-value	Interpretation			
**Acetate**	1,93E-03#	**Ec>Kp> Pa**	5,25E-03#	**Ec>Kp = Pa**	2,43E-03#	**Ec>Kp> Pa**	Ec>Kp>Pa	Ec>Kp>Pa	Ec>Kp>Pa
**Alanine**	9,00E-03#	**Ec>Kp = Pa**	1,93E-03#	**Ec>Kp> Pa**	9,78E-08	**Ec>Kp> Pa**	Ec>Kp>Pa	Ec>Kp>Pa	Ec>Kp>Pa
**Cholate**	1,93E-03#	**Ec> Pa >Kp**	8,02E-03#	**Ec = Pa >Kp**	9,31E-06	**Ec>Kp = Pa**	Ec>Pa>Kp	Ec>Pa>Kp	Ec>Pa>Kp
**Formate**	7,97E-03#	Ec = Pa >Kp	1,71E-02#	Ec = Pa >Kp	8,06E-03#	Ec = Pa >Kp	Ec>Kp>Pa	Ec>Kp>Pa	Ec>Kp>Pa
**Glycine**	3,07E-03#	**Pa >Kp>Ec**	3,07E-03#	**Pa >Kp>Ec**	1,01E-06	**Pa >Ec = Kp**	Pa>Kp>Ec	Pa>Kp>Ec	Pa>Kp>Ec
**Isoleucine**	9,75E-03#	**Inconclusive**	3,03E-03#	**Kp>Ec> Pa**	5,80E-01	**Pa = Kp = Ec**	**Kp>Pa>Ec**	**Kp>Ec>Pa**	**Kp>Ec>Pa**
**Lactate**	3,06E-03#	Ec>Kp> Pa	1,93E-03#	Ec>Kp> Pa	1,93E-03#	Ec>Kp> Pa	Ec>Kp>Pa	Ec>Kp>Pa	Ec>Kp>Pa
**Leucine**	6,81E-03#	**Kp>Ec = Pa**	9,00E-03#	**Pa >Ec = Kp**	3,47E-01	**Pa = Kp = Ec**	Kp>Pa>Ec	Kp>Pa>Ec	Kp>Ec>Pa
**Lysine**	2,99E-02#	**Pa = Kp = Ec**	8,65E-03#	**Ec = Pa >Kp**	4,44E-05	**Ec = Pa >Kp**	Ec>Pa>Kp	Ec>Pa>Kp	Ec>Pa>Kp
**Methionine**	5,85E-03#	**Kp>Ec = Pa**	4,79E-03#	**Pa >Kp>Ec**	8,65E-03#	**Kp>Ec = Pa**	Pa>Kp>Ec	Pa>Kp>Ec	Pa>Kp>Ec
**NAD**	9,00E-09	**Ec> Pa >Kp**	2,49E-01#	**Pa = Kp = Ec**	1,84E-04	**Ec>Kp = Pa**	Ec>Pa>Kp	Ec>Pa>Kp	Ec>Pa>Kp
**o-Phosphocholine**	8,58E-03#	**Kp>Ec = Pa**	9,12E-03#	**Kp = Pa >Ec**	1,83E-02#	**Kp>Ec = Pa**	Pa>Kp>Ec	Pa>Kp>Ec	Pa>Kp>Ec
**Phenylalanine**	1,21E-01#	**Pa = Kp = Ec**	3,46E-02	**Inconclusive**	1,72E-01	**Pa = Kp = Ec**	Pa>Kp>Ec	Pa>Kp>Ec	Pa>Kp>Ec
**Pyruvate**	1,73E-01#	**Pa = Kp = Ec**	3,47E-02#	**Inconclusive**	6,05E-05	**Ec>Kp> Pa**	**Kp>Pa>Ec**	**Ec>Kp>Pa**	**Ec>Kp>Pa**
**Succinate**	8,65E-03#	Kp = Pa >Ec	1,06E-02#	Kp = Pa >Ec	8,65E-03#	Kp = Pa >Ec	Kp>Ec>Pa	Kp>Ec>Pa	Kp>Ec>Pa
**Threonine**	1,22E-02#	**Ec>Kp = Pa**	1,06E-02#	**Ec>Kp = Pa**	2,64E-06	**Ec>Kp> Pa**	Ec>Kp>Pa	Ec>Kp>Pa	Ec>Kp>Pa
**Uracil**	1,93E-03#	**Kp>Ec> Pa**	6,99E-03#	**Kp = Pa >Ec**	4,47E-03#	**Kp = Pa >Ec**	Kp>Ec>Pa	Kp>Ec>Pa	Kp>Ec>Pa
**Valine**	6,81E-03#	**Kp>Ec = Pa**	7,86E-03	**Kp = Pa >Ec**	9,46E-01#	**Pa = Kp = Ec**	**Kp>Pa>Ec**	**Kp>Ec>Pa**	**Ec>Kp>Pa**

-Kruskal-Wallis test; grey background – results with p-value < 0.05; table cells that are in bold and underlined – statistical interpretation or average relation is different for each disintegration method

Table S5. Variation of metabolite concentration between gram-positive bacteria within disruption method.
(Ef – *E. feacalis*; Bc – *B. cereus*; Cg – *C. glutamicum*)

Metabolite	Statistical comparison						Average concentration relation		
	Sonication		Sand mill		Tissue Lyser		Sonication	Sand mill	Tissue Lyser
	p-value	Interpretation	p-value	Interpretation	p-value	Interpretation			
**4-aminobutyrate**	9,19E-03[#]	Ef = Bc>Cg	5,13E-04	Ef = Bc>Cg	8,15E-03[#]	Ef = Bc>Cg	Ef>Bc>Cg	Ef>Bc>Cg	Ef>Bc>Cg
**Acetate**	9,19E-03[#]	Ef = Bc>Cg	6,81E-03[#]	Ef = Bc>Cg	5,25E-03[#]	Ef = Bc>Cg	**Ef>Bc>Cg**	**Ef>Bc>Cg**	**Bc>Ef>Cg**
**Alanine**	9,91E-09	Cg>Bc> Ef	9,36E-06	Cg>Bc> Ef	7,56E-07	Cg>Bc> Ef	Cg>Bc>Ef	Cg>Bc>Ef	Cg>Bc>Ef
**Aspartate**	1,00E-03	**Ef >Bc = Cg**	9,19E-06	**Ef = Bc>Cg**	3,58E-05	**Ef >Bc>Cg**	Ef>Bc>Cg	Ef>Bc>Cg	Ef>Bc>Cg
**Betaine**	3,74E-03[#]	**Bc = Cg> Ef**	4,47E-03[#]	**Bc = Cg> Ef**	1,93E-03[#]	**Bc>Cg> Ef**	Bc>Cg>Ef	Bc>Cg>Ef	Bc>Cg>Ef
**Cholate**	4,04E-03[#]	**Cg>Bc> Ef**	2,61E-02[#]	**Inconclusive**	7,71E-03[#]	**Bc = Cg> Ef**	Cg>Bc>Ef	Cg>Bc>Ef	Cg>Bc>Ef
**Formate**	1,93E-03[#]	Ef >Bc>Cg	3,07E-03[#]	Ef >Bc>Cg	1,93E-03[#]	Ef >Bc>Cg	Ef>Bc>Cg	Ef>Bc>Cg	Ef>Bc>Cg
**Glutamate**	1,28E-07	Cg> Ef >Bc	6,04E-05	Cg> Ef >Bc	4,39E-05	Cg> Ef >Bc	Cg>Ef>Bc	Cg>Ef>Bc	Cg>Ef>Bc
**Glycine**	1,32E-08	**Ef >Bc = Cg**	6,99E-03[#]	**Cg> Ef = Bc**	5,28E-06	**Bc = Cg> Ef**	**Cg>Ef>Bc**	**Cg>Ef>Bc**	**Cg>Bc>Ef**
**Histidine**	1,91E-03[#]	**Cg> Ef >Bc**	3,49E-04	**Ef >Bc = Cg**	2,21E-01[#]	**Cg = Bc = Ef**	**Cg>Ef>Bc**	**Cg>Ef>Bc**	**Bc>Cg>Ef**
**Inosine**	1,12E-02[#]	**Cg = Bc = Ef**	8,15E-03[#]	**Ef = Bc>Cg**	8,65E-03[#]	**Bc> Ef = Cg**	Bc>Ef>Cg	Bc>Ef>Cg	Bc>Ef>Cg
**Isoleucine**	3,12E-04	Ef = Cg>Bc	1,19E-03	Ef = Cg>Bc	1,35E-03	Ef = Cg>Bc	**Cg>Ef>Bc**	**Ef>Cg>Bc**	**Cg>Ef>Bc**
**Lactate**	1,93E-03[#]	Ef >Cg>Bc	3,07E-03[#]	Ef >Cg>Bc	1,80E-06	Ef >Cg>Bc	Ef>Cg>Bc	Ef>Cg>Bc	Ef>Cg>Bc
**Leucine**	1,61E-02[#]	**Inconclusive**	4,35E-04	**Ef = Cg>Bc**	3,93E-02	**Inconclusive**	**Cg>Ef>Bc**	**Ef>Cg>Bc**	**Cg>Ef>Bc**
**Lysine**	8,10E-01[#]	**Cg = Bc = Ef**	4,52E-04	**Ef = Bc>Cg**	2,21E-01	**Cg = Bc = Ef**	**Ef>Bc>Cg**	**Ef>Bc>Cg**	**Bc>Ef>Cg**
**Methionine**	4,47E-03[#]	**Bc = Cg> Ef**	7,23E-06	**Cg>Bc> Ef**	8,06E-03[#]	**Bc = Cg> Ef**	**Cg>Bc>Ef**	**Cg>Bc>Ef**	**Bc>Cg>Ef**
**NAD**	1,11E-02	Inconclusive	3,21E-02	Inconclusive	9,75E-03[#]	Inconclusive	**Bc>Cg>Ef**	**Bc>Ef>Cg**	**Bc>Cg>Ef**
**o-Phosphocholine**	3,74E-03[#]	**Ef >Bc = Cg**	9,19E-03[#]	**Ef >Bc = Cg**	1,93E-03[#]	**Ef >Bc>Cg**	Ef>Bc>Cg	Ef>Bc>Cg	Ef>Bc>Cg
**Phenylalanine**	7,73E-07	**Cg>Bc> Ef**	3,20E-04	**Cg> Ef = Bc**	5,44E-05	**Cg> Ef = Bc**	Cg>Bc>Ef	Cg>Bc>Ef	Cg>Bc>Ef
**Pyruvate**	3,03E-03[#]	Cg> Ef >Bc	7,56E-08	Cg> Ef >Bc	1,38E-09	Cg> Ef >Bc	Cg>Ef>Bc	Cg>Ef>Bc	Cg>Ef>Bc
**Sarcosine**	2,01E-03	**Ef = Bc>Cg**	4,17E-01	**Cg = Bc = Ef**	6,04E-03[#]	**Bc> Ef = Cg**	**Ef>Cg>Bc**	**Bc>Ef>Cg**	**Bc>Cg>Ef**
**Succinate**	1,93E-03[#]	Cg>Bc> Ef	1,93E-03[#]	Cg>Bc> Ef	1,93E-03[#]	Cg>Bc> Ef	Cg>Bc>Ef	Cg>Bc>Ef	Cg>Bc>Ef
**Threonine**	2,64E-08	Cg> Ef >Bc	1,05E-07	Cg> Ef >Bc	1,95E-07	Cg> Ef >Bc	Cg>Ef>Bc	Cg>Ef>Bc	Cg>Ef>Bc
**Tyramine**	2,04E-05	**Cg> Ef >Bc**	1,06E-05	**Ef = Cg>Bc**	5,05E-05	**Ef = Cg>Bc**	Cg>Ef>Bc	Cg>Ef>Bc	Cg>Ef>Bc
**Tyrosine**	3,48E-07	Cg> Ef >Bc	6,04E-06	Cg> Ef >Bc	1,06E-05	Cg> Ef >Bc	Cg>Ef>Bc	Cg>Ef>Bc	Cg>Ef>Bc
**Valine**	5,43E-06	Ef = Cg>Bc	1,54E-05	Ef = Cg>Bc	4,41E-05	Ef = Cg>Bc	**Cg>Ef>Bc**	**Ef>Cg>Bc**	**Cg>Ef>Bc**

[#] - Kruskal-Wallis test; grey background – results with p-value < 0.05; table cells that are in bold and underlined – statistical interpretation or average relation is different for each disintegration method

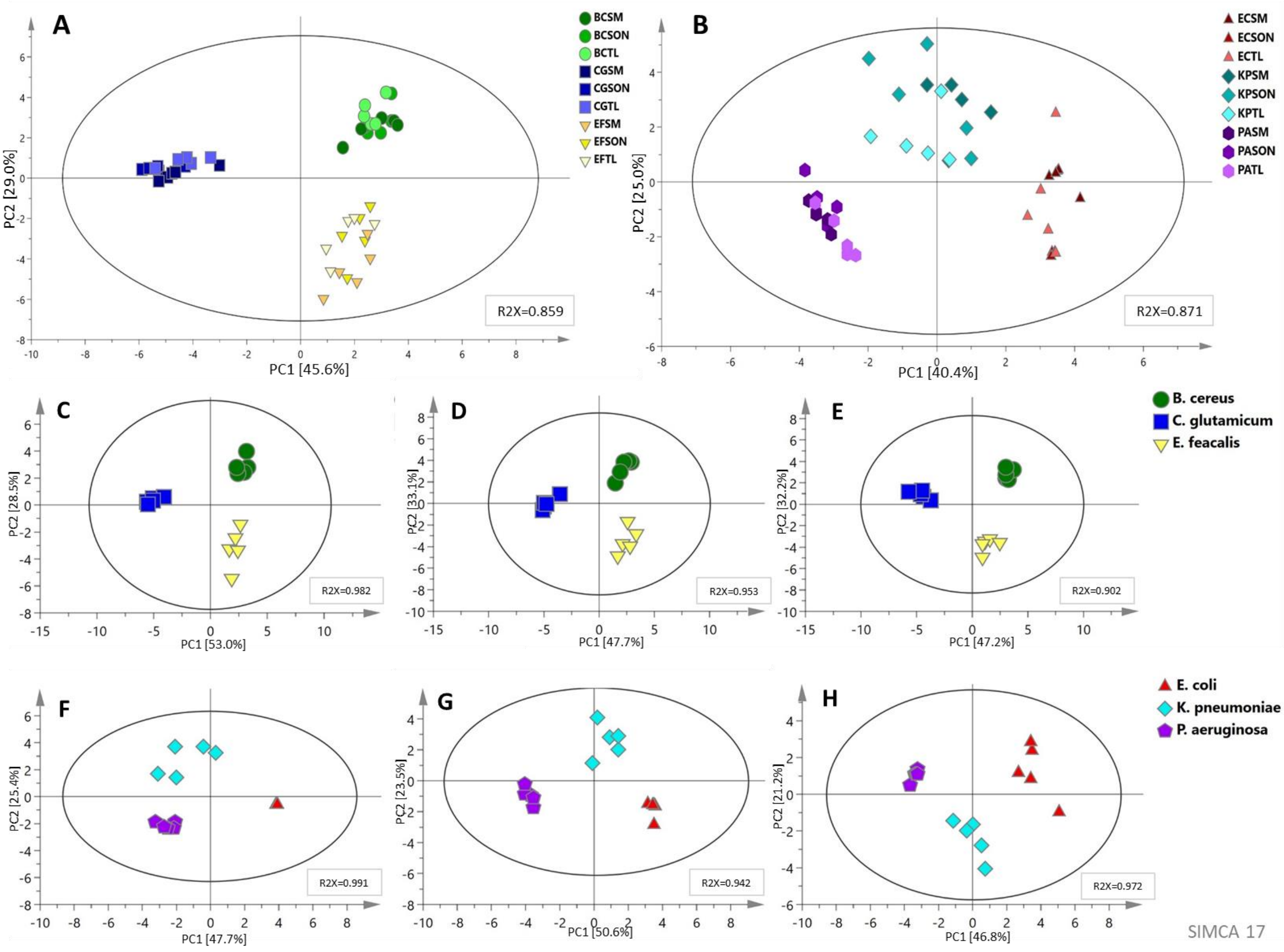

Figure S4. PCA score plots for separate analysis of gram-positive and gram-negative strains. A – gram-positive strains, all disintegration methods; B – gram-negative strains, all disintegration methods; C - gram-positive strains, sonication; D - gram-positive strains, sand mill; E - gram-positive strains, tissue lyser; F - gram-negative strains, sonication; G - gram-negative strains, sand mill; H - gram-negative strains, tissue lyser.

((BCSM* – B. cereus *(sand mill); BCSON* – B. cereus *(sonication); BCTL* – B. cereus *(tissue lyser); CGSM* – C. glutamicum *(sand mill); CGSON* – C. glutamicum *(sonication); CGTL* – C. glutamicum *(tissue lyser); ECSM* – E. coli *(sand mill); ECSON* – E. coli *(sonication); ECTL* – E. coli *(tissue lyser); EFSM* – E. feacalis *(sand mill); EFSON* – E. feacalis *(sonication); EFTL* – E. feacalis *(tissue lyser); KPSM* – K. pneumoniae *(sand mill); KPSON* – K. pneumoniae *(sonication); KPTL* – K. pneumoniae *(tissue lyser); PASM* – P. aeruginosa *(sand mill); PASON* – P. aeruginosa *(sonication); PATL* – P. aeruginosa *(tissue lyser))
